# Editorial: Current knowledge on camelids infectious and parasitic diseases

**DOI:** 10.3389/fvets.2023.1351318

**Published:** 2024-01-04

**Authors:** Abdelmalik Ibrahim Khalafalla, Maryam Dadar, Alireza Sazmand

**Affiliations:** ^1^Biosecurity Affairs Division, Development, and Innovation Sector, Abu Dhabi Agriculture and Food Safety Authority (ADAFSA), Abu Dhabi, United Arab Emirates; ^2^Agricultural Research, Education and Extention Organization (AREEO), Razi Vaccine and Serum Research Institute, Karaj, Iran; ^3^Department of Pathobiology, Faculty of Veterinary Medicine, Bu-Ali Sina University, Hamedan, Iran

**Keywords:** editorial, current knowledge, camelids, infectious diseases, parasitic diseases

We invited camelid scientists across the globe to submit their research on camelid diseases to highlight the importance of diseases of camelids, including Old World camels (OWC; one-humped dromedary and two-humped Bactrian camels) and New World camels (NWC; llama, alpaca, guanaco, vicuna) also known as South American camelids (SAC). We welcomed submissions in the broad subject area of endo- and ectoparasitic, bacterial, viral, and fungal infections of old and new world camelids. Finally, we accepted 10 articles on dromedaries (*n* = 7), Bactrians (*n* = 2), and NWCs (*n* = 1) written by 83 authors from 11 countries.

Tick infestation of camels is a significant challenge, impacting not only their productivity and wellbeing but also several pathogens they may transmit, some of which are zoonotic. Although chemical acaricides have been the primary means of controlling ticks, there is growing interest in developing environment-friendly herb-based acaricides. In this Research Topic, a groundbreaking study by Gareh et al. explored the acaricidal potential of neem seed extracts (*Azadirachta indica*) on the camel tick *Hyalomma dromedarii*, which is considered the most prevalent tick-infesting dromedaries. The hexane extract was the most effective, showing 100% tick mortality within 1 day at a 20% concentration. This research points to a natural alternative for tick control and emphasizes the economic viability of using neem seed extracts.

Intestinal protozoan parasites of dromedary camels have yet to be extensively studied to clarify their occurrence, diversity, and zoonotic potential since most of the research on the subject has relied on microscopic examination of fecal material. In this Research Topic, Elmahallawy et al. investigated PCR positivity of camels' fecal specimens in Egypt for *Cryptosporidium* spp., *Giardia duodenalis*, and *Enterocytozoon bieneusi*. Although *E. bieneusi* was not detected, identification of zoonotic *C. parvum, C. bovis* and *Giardia duodenalis* implies the role of camels as sources of oo (cyst)/spore contamination in the environment. In this article global occurrence and genetic diversity of *Cryptosporidium* spp., *Giardia duodenalis*, and *E. bieneusi* reported in OWCs is summarized in a table.

The protozoan parasite *Neospora caninum* which infects many species of warm-blooded animals is a major cause of bovine abortion worldwide. Although there is lots of information about neosporosis of dromedaries, there is shortage of knowledge regarding Bactrians exposed to *N. caninum*. In the first epidemiological study on neosporosis in camels in China, Qi et al. examined serum samples from nine animal species including *Camelus bactrianus* by indirect ELISAs detecting *N. caninum*-specific IgG and IgM antibodies. The findings suggested significant exposure, emphasizing the need for further exploration into the role of different animals in the epidemiology of this ubiquitous parasite.

Eimeriosis is an economically important parasitic disease in all camelid species. In an article from Mongolia, Khatanbaatar et al. examined 536 fresh fecal samples from Bactrians to identify *Eimeria* parasites diversity, then screened the genetic diversity in a functional important immune response gene of the major histocompatibility complex (MHC). This research not only identifies *E. cameli, E. rajasthani* and *E. dromedarii* but also delves into the immunogenetic response of infected and non-infected camels. Understanding the host-parasite interactions is crucial for developing effective strategies against this parasitic disease.

Mastitis, an inflammatory condition of the mammary gland, one of the most significant infectious diseases affecting camels, causes substantial financial losses since it lowers the quantity and quality of milk produced. Bacterial infections are a common cause of mastitis in these animals. The primary pathogens involved in camelid mastitis include *Staphylococcus aureus, Streptococcus* species, and *Escherichia coli*. An efficient immune response to mastitis pathogens depends on the mammary glands' resistant cell makeup and function being in balance. In this Research Topic, mastitis is explored at the cellular level by Alhafiz et al. The study reveals changes in immune cell composition and function in camels with subclinical mastitis. These insights into the mammary gland's immune response contribute to a better understanding of host-pathogen interactions.

Globally, tuberculosis (TB) is a significant public health concern, particularly in developing countries with tropical climates. However, there are very few reports of congenital tuberculosis in people and animals. Narnaware et al. reported congenital TB caused by *M. tuberculosis* in a dromedary camel fetus with a possible vertical transmission. Authors suggested regular screening of camels for mycobacterial infection to minimize the risk associated with the spread of TB in endemic areas.

The study of coronaviruses has grown significantly in recent years. MERS-CoV replicates in various cell types, and quick development has been made of assays for its growth and quantification. However, only a few viral isolates with complete characterization are available for investigation. Middle East respiratory syndrome coronavirus (MERS-CoV) has been a focus of global health concern. Khalafalla et al. isolated and genetically characterized MERS-CoV from Dromedary Camels in the UAE in this Research Topic. The isolates contained several amino acid substitutions, and the analysis further identified a recombination event in one of the reported sequences. The findings underscore the importance of continuous monitoring and characterization of MERS-CoV for effective control measures.

Camels have been long implicated in transmitting various zoonotic diseases but the discovery of MERS-CoV sparked interest in camels as reservoirs of zoonotic pathogens. Khalafalla reviewed the literature on zoonotic diseases transmitted from camels, focusing on those with epidemiological or molecular evidence of transmission from camels to humans. This comprehensive review highlights diseases such as MERS, brucellosis, and anthrax, emphasizing surveillance, preventive measures, and a one-health approach to mitigate risks.

Apart from the pathogens and diseases, one of the most critical aspects of infection prevention and animal disease control is livestock farmers' Knowledge, Attitude and Practices (KAP). In the heart of camel-keeping regions in Kenya, the KAP survey by Othieno et al. sheds light on the challenges faced by camel herders. Respiratory diseases, indiscriminate drug use, and various constraints are identified, emphasizing the need for targeted interventions and heightened awareness. According to the authors, watering points, grazing areas, and marketing points are the primary areas for congregating camels and have a significant potential for disease spread.

Although the husbandry of South American camelids (SAC) is becoming more popular and different diseases and conditions cause emaciation, there is lack of a standardized guideline for the body condition score (BCS) in llamas or alpacas. Wagener et al. evaluated comparability of BCS assessment of six examiners including veterinarian, veterinary student and animal keeper given to 20 llamas and nine alpacas in Germany. The findings highlight the reliability of palpation of the lumbar vertebrae as a method for determining nutritional status in llamas and alpacas and that reproducibility increases with training and experience.

In conclusion, these articles collectively contribute to our understanding of camel health, revealing insights into camel health from the molecular world of parasites to the broader context of zoonotic risks. The findings underscore the importance of comprehensive research, surveillance, and awareness to ensure the wellbeing of camels and mitigate potential health risks to humans.

## Author contributions

AK: Writing – original draft, Conceptualization. MD: Writing – review & editing, Conceptualization. AS: Writing – review & editing, Conceptualization.

